# ‘Creative environmental engineering’ and the cost of environmental claims: legitimisation of tire artificial reefs by US federal scientists in 1960s–1970s

**DOI:** 10.1007/s40656-025-00700-7

**Published:** 2025-12-17

**Authors:** D. Porsnovs

**Affiliations:** The Greenhouse Centre for Environmental Humanities, University of Stavanger, Stavanger, Norway

**Keywords:** Artificial reefs, Environmental claims, Sandy hook marine laboratory, Social constructionism, Tires, Waste

## Abstract

Between 1966 and 1974, the US Bureau of Sport Fisheries and Wildlife carried out research on the possibilities of using waste materials for constructing artificial reefs. One of the main outcomes was the recommendation to construct reefs from waste tires. With the encouragement of the findings, more than 700 reefs containing tens of millions of tires were built along the US Atlantic seaboard. This article investigates the artificial reef study at Sandy Hook Marine Laboratory and analyses environmental claims built on its results. The results show that fisheries scientists involved in artificial reef research framed tire artificial reefs as a solution to two problems: recreational fishing enhancement and waste disposal. Their claims about the appropriateness of tire reefs became a driving force of their construction in the US.

## Introduction

The Goodyear Corporation’s blimp glides across the golden-hour-tinted sky of Southern Florida as a professional voice behind the scenes says:

The airship Mayflower, halfway to Marathon in the Florida Keys. It’s the eve of a very special day. Before another sunset, Marathon residents will see the culmination of many years of planning and hard work. They will be building an artificial reef made of discarded tires.

This professionally shot promotional film called *D-day in Marathon*, produced for the Goodyear corporation (McGowan, 1977), tells the story of the “drop day” for making an artificial reef for fishing improvement from waste tires in 1977. The film explains:

Finally, D-day, the dreams-come-true day, the day of the first tire drop, laying the reef’s cornerstone… It’s an opportunity for them to improve their sports fishing. Equally important, the reef should be good for the tourist-based economy. At the same time, they are ridding the Keys of some accumulated trash. Call it creative environmental engineering.

The film shows hundreds of smiling people working to solve two serious problems: diminishing fish populations for recreational fishing and escalating accumulation of waste tires overflowing landfills. The film’s narration states, “It is a real community effort, like an old-fashioned barn raising. You will find doctors, lawyers, merchants, chiefs, parents, teachers, kids”. Everybody seemingly worked together—even one chimpanzee was seen carrying tires to the boat—with many organizations taking part: Marathon Rotary Club served breakfast for the participants and coordinated the project, the US Coast Guard regulated the traffic of the boat armada, and the US Navy provided their landing ship to transport the tires to the reef site. All the festivities were led from the sky by the Goodyear blimp. The film unequivocally shows the collective conviction of these individuals, who are confident that their endeavors contribute positively to the marine environment and their local community.

*D-day in Marathon*’s overt invocation of World War II’s D-Day and the Allied Powers’ victory hints at the seriousness of the problem they attempted to tackle. In the latter half of the twentieth century, coastal communities in the United States and beyond began grappling with the repercussions of coastal development and the earlier destruction of estuaries. The result was diminishing fish populations, occurring simultaneously with increased recreational fishing (McClenachan, 2009). During this era, fishing for fun had already evolved into a well-established industry bearing both cultural and economic significance. By 1955, approximately 4.5 million saltwater fishermen had collectively injected roughly one billion dollars into the U.S. economy. Notably, by 1965, the number of anglers had surged to over 8.3 million, and the economic value of the recreational fishing industry significantly eclipsed that of commercial fishing (De Sylva, 1969). The profound desire of people to fish compelled them to seek remedies for the declining fish populations, with artificial reefs emerging as a promising solution. This was the context for Marathon’s D-Day. In this article, I investigate the thinking behind artificial reefs built of waste tires and how they were promoted as a solution for two separate environmental problems faced by the US in the 1960s and 1970s: waste accumulation and fishing declines.

Waste history has emerged as a vital subfield within environmental and urban studies, examining how societies have managed and perceived waste over time. This field investigates waste policies’ cultural, economic, and technological dimensions and their influence on urban landscapes and public health. Pioneers like Martin Melosi (1981, 2000) laid the foundation with his analysis of the evolution of waste management systems and their lasting legacies. Subsequent contributions, including Carl A. Zimring’s (2005, 2017) research on metal recycling and Marco Armiero’s (2021) concept of the Wasteocene, broadened the field by integrating themes of environmental justice and socio-political inequalities. Recent interdisciplinary approaches, such as discard studies, have emphasized waste as a lens for critiquing power dynamics and societal values, highlighting its central role in shaping modern environmental and social narratives (Liboiron & Lepawsky, 2022; Moore, 2012). The case presented here is particularly significant in waste history, as it challenges the traditionally clear ontological boundaries between waste and non-waste, demonstrating how environmental claims-making can reshape these boundaries.

Waste history has frequently intersected with water history, as water has often been perceived as the ultimate sink for various waste products generated by human economies (Tarr, 1996). Oceans have served as dumping grounds for hazardous materials, including chemical weapons (Dawson, 2023) and high-activity radioactive waste (Hamblin, 2008). Civic opposition to ocean waste disposal has been crucial in developing the Western environmental movement (Fazzi, 2023). However, historians have not analyzed instances where ocean waste disposal was considered beneficial for the ocean and organisms living there.

Histories of waste management practices intersect in this study with research on histories of overfishing and the depletion of marine populations. Studies highlight how centuries of industrial exploitation, resource-centered management, and shifting baselines have driven marine population declines while underscoring the need for systemic change in how humans interact with ocean ecosystems (Roberts, 2007; Rose, 2007; Telesca, 2020). However, subsistence and recreational coastal fisheries, often perceived as low impact, have received comparatively less attention. McClenachan (2013) challenges this narrative by showing how recreational anglers have substantially reduced vulnerable nearshore fish populations and obstructed effective conservation policies. Studying coastal fisheries, their problems, and the solutions chosen to overcome them are crucial for understanding marine exploitation’s broader ecological and cultural implications.

Fisheries scientists and oceanographers shape the interactions between the ocean environment and humans. Sociologist Michel Callon (1986) described how marine biologists tried to fight the decline in the population of scallops in St. Brieuc Bay and conceptualized their actions as the four stages of the translation, a particular form of network interaction in which one member acts on behalf of the others and the process through which this representation is established and eventually dissolved. Environmental historian Dolly Jørgensen (2009) similarly analyzed the scientific and public discourse surrounding the Gulf of Mexico rigs-to-reefs program, illustrating how various groups constructed the view of petroleum platforms as a necessary part of the Gulf of Mexico ecosystem and how these activities altered platform removal policies. Historian of science Julija Lajus (2018) has examined the Barents Sea herring fisheries, illustrating how the Soviet government, during the 1930s, constructed a resource that did not exist in nature through the intermediary role of scientists. They have demonstrated that scientists represent marine organisms and their interests and these representations shape policies and collective action.

Fisheries scientists have frequently approached their field with a resource-centered perspective, treating fish populations as discrete entities to be studied and optimized for harvest. Throughout the twentieth century, the professionalization of fisheries science led to the gradual replacement of dynamic, ecosystem-oriented approaches with more narrowly focused, resource-centric paradigms (Hubbard, 2006). The development of concepts such as Maximum Sustainable Yield (MSY) reflects this reductionist approach, prioritizing the maximization of yields over the maintenance of ecological resilience. As Finley (2011) highlighted, this resource-focused orientation within fisheries science often sidelined ecological considerations, contributing to unsustainable practices and the overexploitation of marine resources. Scientific views about fish as resources have had a direct effect on policy development (Telesca, 2020).

When scientists put forward understandings of the world, particularly in public spaces and settings, they are making claims. An environmental claim represents a specific case of discursive construction, where the claims-maker asserts the environmental consequences of a particular anthropogenic impact. Typically, such claims characterize an action or phenomenon as beneficial or harmful to the environment. The environmental claims-making conceptual framework originates from social-constructivist approaches in environmental sociology. Sociologist Joel Best (2017) has proposed that the key questions to be considered when analyzing the content of a claim are: What is being said about the problem? and, what is the rhetoric of claims-making—how are claims presented to persuade their audiences? Environmental sociologist Hannigan (2022) has identified six prerequisites for successfully constructing an environmental problem. According to him, in the dynamics of environmental discourse, the validation of claims relies heavily on scientific authority, ensuring a robust foundation for assertions. Individuals known as popularizers play a critical role in navigating the intersection of environmentalism and scientific domains and facilitating effective communication. Media attention strategically shapes the narrative, framing the identified issue as distinctive and crucial. The dramatization of the problem is achieved through symbolic and visual representation, capturing the attention and concern of the wider audience. Economic incentives emerge as a driving force, encouraging proactive measures to address environmental challenges. Furthermore, enlisting an institutional sponsor becomes pivotal, contributing to the cause’s legitimacy and ensuring a sustained commitment to addressing the identified issue. Unpacking scientists’ environmental claims is a particularly fruitful approach to analyzing how the scientific discourse was constructed and exerted influence.

Environmental claims, including misleading ones often called greenwashing, have garnered significant attention in communication, marketing, and advertising studies (Baum, 2012; Netto et al., 2020; Phau & Ong, 2007; Shahrin et al., 2017). Scholars from interdisciplinary studies of the environment have also used the framework. For example, Emma Jakku (2009) applied it to scrutinize the process of the construction of an environmental problem by analyzing the case of the issue of sand and gravel extraction from the Brisbane River, and A. Hanson (2000) analyzed how British mass media used framing in their ideological work during the coverage of the Brent Spar ocean dumping controversy. Geographers Carol Morris & Amanda Wragg (2003) have studied the role of non-media claims-makers in legitimizing biodiversity as the primary concern during the implementation of Biodiversity Action Plans in the UK. The environmental claims-making approach slightly differs from a typical Actor-network theory analysis that emphasizes the significance of actor enrollment in historical processes (Latour, 2007). Instead, this method focuses on the origin of claims and identification of the claims-makers. Analysis of the mechanisms of the social construction of environmental knowledge enables scholars to interrogate how historical and cultural contexts produce and shape our understanding of the natural world (Prichard 2013).

Using the claims-making approach, geographer Meindl and his colleagues conceptualized applied scientists as environmental claims-makers who give technical assessments of nature and transform this knowledge into practices that become accepted and applied in public. Their article analyzed claims-making activities related to Florida Everglades’ wetlands by engineer James O. Wright at the beginning of the twentieth century. Despite the claims being misleading, Wright’s work and his claims converted technical ideas into practices promoting large-scale drainage of the Everglades (Meindl et al., 2002). Meindl’s approach acts as a model for applying the environmental claims concept in historical research.

Building on environmental claims scholarship, this article analyses the activities of the scientists who worked for the federal artificial reef research program between 1966 and 1975. It examines how they described environmental problems, their apparent solutions by making environmental claims, and how these claims were enacted as collective action. Building on congressional documents, reports, public communication of the group, and mass-media materials about artificial reef building, this article argues that the claims-making by a group of federal fisheries scientists employed by USFWS directly influenced the construction of tire reefs in the second half of twentieth century all along the East Coast of the US.

## Artificial reef context

Artificial reefs are human-made structures designed to replicate specific characteristics of natural reefs, serving as effective tools for enhancing marine productivity and fishery resources. By introducing solid substrates into ocean areas lacking natural surfaces, these structures provide attachment points for marine plants and organisms, thereby fostering ecosystem development. In nutrient-rich but substrate-deficient environments, artificial reefs create essential habitats that support diverse marine life. They attract fish and other organisms by offering food, shelter, and protection while also influencing water flow and nutrient circulation. Sometimes artificial reefs evolve into thriving habitats that support a wide range of species, including juvenile fish that benefit from increased protection and reduced predation risks (Layman & Allgeier 2020; Vivier et al. 2021). The primary goal of constructing an artificial reef is often to create a habitat abundant in food resources, thereby attracting large predatory fish such as black sea bass or red snapper to locations known to fishermen.

The earliest documented artificial reef-building effort in the US occurred in the second half of the 19ts century when Florida fishermen sank logs into coastal waters to compensate for the destruction of natural mangrove habitats caused by cotton cultivation (Elliot, 1867). At the beginning of the twentieth century, groups of fishing boat owners started more organized efforts to create more productive fishing grounds for their clients. For example, in the middle 1920s, New York’s Boatmen’s Association created some reefs in the Great South Bay by sinking wooden butter tubs filled with concrete (New York State Department of Environmental Conservation 1993). The 1930s marked a shift toward the use of waste, with the deployment of cement-filled drums, old automobiles, and concrete rubble near the New Jersey coast (Anonymous, 1966). World War II interrupted these efforts, as gasoline shortages and wartime threats curtailed recreational fishing. However, numerous unintentional reefs emerged from military shipwrecks and life-fire drills (Hutt, 1961; NOAA Office for Coastal Management, 2022).

Postwar America saw a resurgence in reef building, driven by the dramatic rise in recreational fishing participation and economic impact. Fantastic fishing on a wartime shipwreck in the vicinity of Orange Beach, Alabama, inspired the local fishermen to use waste materials, such as old cars, to build new fishing grounds, culminating in large-scale deployments by the Orange Beach Deep-Sea Fishing Association (Allen, 1970). Significant artificial reef building activities took place in New York, where waste disposal challenges were transformed into highly productive fishing grounds by well-planned dumping of demolition rubble and some industrial waste in the bays (Buchanan et al., 1988; Stroud, 1961). In Texas, oyster shell reefs and later robust fish havens built from concrete and steel reinforced the state’s coastal fishing infrastructure (Anonymous, 1964, Texas A&M University 1974).

The 1950s and 1960s brought a surge in artificial reef projects, bolstered by federal support through the Sport Fish Restoration Program, which channeled funds from excise taxes on fishing gear (Stroud, 1961; Massmann 1976). These decades also saw the first attempts of systematic scientific research into artificial reefs, exemplified by John Randall’s pioneering study in the Virgin Islands (Randall, 1963). Meanwhile, the oil industry’s offshore platforms along the Louisiana coast inadvertently became some of the most extensive artificial reef systems, fostering rich marine ecosystems (Wilson, Van Sickle & Pope 1987; Jørgensen, 2009). By the 1960s, artificial reef building was firmly established along the Atlantic coast of the U.S. to allow the local communities to improve fishing in the areas accessible by small boats. Yet, the scale of these activities was small before the 1970s.

## Fishery science linking waste and reefs

Established in 1960 as a vital component of the federal Marine Game Fish Research program of the USFWS, the Sandy Hook Marine Laboratory in New Jersey was a cutting-edge marine biology research center. Throughout the 1960s, the laboratory conducted monthly surveys of the Atlantic continental shelf to map the seasonal distribution of game fish eggs, larvae, and juveniles, particularly in estuarine areas. The laboratory pioneered methodologies for studying the daily movements and feeding behavior of game fish in their natural habitat using SCUBA diving (US House of Representatives, 1966).

In the mid-1960s, scientists at Sandy Hook discovered that fluke fish migrate annually. To enhance the population of these fish, researchers proposed strategically placed artificial reefs along their migration route. The original concept involved repurposing old car bodies to create a chain of 12 artificial reefs spanning the Atlantic coast, from Connecticut to Florida, which would serve as feeding stations to improve the survival rates of fluke. Their proposal to create artificial reefs from discarded materials tapped into contemporary concerns about waste disposal and potential funding opportunities (US Senate, 1966). Following World War II, the United States witnessed rapid economic development, leading to increased affluence and significant environmental problems, including pollution, habitat loss, and a growing volume of waste. The latter part of the 1960s represented a pivotal moment in American environmental awareness and marked the inception of the environmental movement. Waste management became a subject of substantial regulation with enacting the Solid Waste Disposal Act of 1965, which provided the legal framework for researching and implementing new technologies in this field. Lady Bird Johnson, the wife of President Lyndon B. Johnson, led a beautification initiative with one of the objectives to reduce the number of abandoned cars scattered along American roads and streets. The idea of the artificial reef research program was related to Johnson’s efforts for highway beautification since it was developed to utilize car bodies (Shenfeld, 1970).

In March 1966, Congress conducted an appropriations subcommittee hearing that deliberated on the budget allocations for the artificial reef research program. Lionel Walford, the director of Sandy Hook Marine Laboratory, presented a written statement to Congress that aligned the objective to boost fishing with the necessity to manage waste:

Growing piles of junk cars are a blight upon the country. So are other solid wastes such as building rubble obsolete barges, ships, subway, and railroad cars. Ironically these solid wastes could made to have a constructive rather than a destructive effect by systematically dumping them into the sea in appropriate places to create new habitats for fish and hence artificial fishing reefs. New habitat is desperately needed to improve fishing because large sections of the shallow water environment are being destroyed, partly to make dumping grounds for solid wastes. (US Senate, 1966, p. 2083).

In this statement, Walford made it clear that he viewed artificial reef building as a solution to reach a state that 30 years later was defined by the engineer Robert Frosch (1996) as the “End of Waste”: a situation when the waste loses its harmful properties and again becomes a resource creating value for the economy.

The recreational fishing industry actively lobbied for this funding allocation. During his testimony at the Congressional hearing, Richard C. Wolf, the Association of Marine Angling Clubs leader and the vice president of Garcia Corp (the largest fishing gear company at the time), advocated for increased spending on marine game fishing research. “Now we have a tremendous need for increased recreational facilities. We have a growth of 500,000 fishermen a year and are trying to supply fish for these fishermen” (US Senate, 1966, p.1964). Wolf contended that constructing artificial reefs from waste materials was an innovative approach that would both create fishing habitats and contribute to cleaner American streets.

These artificial reefs serve a twofold purpose. They will give us a chance to get rid of the 2 million ruined autos lying around and on America’s scenery and we don’t know what to do with them. It will allow us to take those million automobiles at the rate of possibly 1 million a year and dump them in the areas designated throughout the entire United States in the eventual program and produce good fishing, produce recreation serving a twofold purpose. (US Senate, 1966 p. 1964)

Both Walford and Wolf emphasized the proposed solution’s ability to solve two problems simultaneously. They used metaphors to persuade Congress to provide funding: Walford characterized the junked autos as a ‘blight’ (a deadly fungal plant disease), and Wolf called the proposed solution’ burial at sea’ and ‘an underwater cemetery with an inexhaustible capacity’, promising to get rid of this huge and ugly problem once and forever.

Although waste management problems might seem far from the sphere of interest of fishery scientists, in this case, the environmental crisis of the 1960s reshaped how waste was viewed in American society. Concepts like recycling, recovery, and reuse gained increased prominence as strategies to conserve natural resources and diminish the reliance on landfills (Brownell, 2011; Melosi, 2004). In this context, it becomes evident that the idea of making something useful of waste appeared remarkably innovative and forward-thinking when presented.

The scientists’ strategy was efficient enough, and congressional budgetary allocation for artificial reef research was granted (*New York Times*, 1972b; Shenfeld, 1970). Young oceanographer Richard Stone, who started his career at Sandy Hook in 1964, was appointed the head of artificial reef research (Stone, n.d.). The aims and objectives of the program were very practical and utilitarian: it aimed to create a set of research reefs along the Atlantic coast. The program would address various questions that state agencies and other organizations involved in reef construction might encounter, including the suitable materials for reef construction, the associated costs, the most effective reef types, and how these reefs could be utilized for resource management (Stone, 1974). It was a typical example of “mandated science” as defined by Salter et al. (1988): the research intended to create standards and a basis for public policy.

## Choosing tires and making claims

Stone’s team constructed its inaugural artificial reef composed of 16 discarded car bodies in 1966 off the coast of Monmouth Beach, New Jersey. The biological processes were studied through monthly SCUBA diver observations and fish counts (Ogren, 1967; Stone, 1972). The first progress report, authored by fisheries scientist Larry Ogren, furnishes a comprehensive account of the ecosystem’s development on the reef. The reef’s capacity to attract fish became conspicuous soon after the community of encrusting organisms became established with a minimum of 11 fish species documented in the vicinity of the reef (Ogren, 1967). The following year, reefs were started in Atlantic Beach (NY), Kiawah Island (SC), Jacksonville Beach (FL), and Biscayne Bay (FL) (Stone et al., 1968). In addition to repurposing automobile bodies, scientists experimented with alternative waste types. At Monmouth Beach, old tires were arranged in groups of twelve, spaced on reinforcing metal rods, and weighted down with concrete. Black sea bass quickly inhabited the tire reef (Stone et al., 1968). Based on data from fouling organism sampling, the team concluded in 1968 that “rubber is the most suitable substrate for colonization by most invertebrate organisms” (Stone et al., 1968, p.179).

In the 1970s, there was a notable surge in car usage in the United States, resulting in a corresponding increase in discarded tires. Despite constituting a small fraction of solid waste, tires posed a persistent challenge due to their resistance to natural decomposition. Their prolonged environmental impact became a growing concern. Incineration faced obstacles, as available pollution control technologies were not cost-effective in meeting new stringent air pollution regulations. Landfill disposal was also problematic, as tires resiliently reverted to their original shape after compaction, resurfacing as the fill settled. Old tires turned into breeding grounds for vermin, mosquitoes, and other disease vectors (Kiefer, 1974). In 1968 alone, the United States generated approximately 3 million tons of discarded tires, underscoring the pressing national imperative to devise innovative methods for the secondary utilization of tires (Pettigrew, 1971). Both governmental authorities and tire manufacturers demonstrated a keen interest in pioneering new approaches to alleviate this pervasive nuisance.

The interpretative claim of Stone’s team that tire rubber was “the *most* suitable substrate” (emphasis added) centered around the acceptability, appropriateness, and even the preference for using tires as reef-building material. The roots of this claim can be traced back to 1967, shortly after the team obtained positive Multiple Disc Sampling Apparatus (MDSA) results of larval settlement on rubber. The MDSA device featured standard discs crafted from a single material (rubber, concrete, steel, etc.), which were removed monthly from the test sites. This method provided insights into the timing of larval settlement, their growth rate, survival, competition, and the progression of species over time. The initial MDSA results revealing successful larval settlement on tire rubber marked a turning point for the researchers, prompting them to favor tires over other materials like automobile bodies that they had also been testing. John Clark, the assistant director of the Sandy Hook laboratory, made the group’s earliest statement recommending the utilization of tires for reef construction during an interview with a Florida fishing journalist: ‘Tires have long life underwater as compared to old auto bodies, which last only three to five years. They also cost less to move and install than auto bodies, rubble, rocks, and cement fish houses’ (Buckow, 1967b). In his statement, the non-biodegradability and stability of tires were seen as an asset that would facilitate marine growth. As a result, the team swiftly shifted their focus from using car bodies to adopting tires as the preferred material for constructing artificial reefs.

Research on tires became the highest priority. In 1968, the National Marine Fisheries Service established a collaborative agreement with the Solid Waste Management Program of the US Environmental Protection Agency (EPA) and the National Tire Dealers and Retreaders Association (NTDRA). This partnership marked the commencement of a comprehensive investigation focused on the tires for constructing reefs (Stone et al., 1974). The initiative also received funding from the New York State Conservation Department (Buckow, 1969a). This indicates that the successful outcomes of the tire experiments attracted the interest of organizations involved in or concerned with tire waste management, and the researchers effectively leveraged this interest to secure additional funding for their work.

Yet after a year of additional study, MDSA results showed that rubber performed below expectations. Concrete proved superior for supporting the growth of a sessile community, while automobile bodies were less effective than rubber (Stone et al., 1969, p. 21). Despite the findings favoring concrete, the significance of this discovery was somewhat overshadowed by the substantial involvement of numerous organizations, specifically the EPA and the NTDRA, who were interested in finding a use for tires, which led the scientists to continue to stress the value of tires. In 1969, team leader Stone stated: “Our experience shows scrap tires make the best reef material in many areas. Tires are easy to obtain because they are a nuisance to dispose of on land but are easy to handle and can be formed into units of almost any size.” (Stone et al., 1969, p. 18). This statement solidified the claim’s status and propelled it forward.

The project team emphasized that the reefing modules needed to have necessary relief, be easy to handle, and be cost-effective. Two types of tire modules—10-tire modules with metal rods and single-tire modules—were recommended to reef builders (Stone & Buchanan, 1970). Combining these modules, the research team deployed 30,000 tires on the Atlantic Beach reef, while 5,000 were placed on the newly established one near Sea Girt (Stone et al., 1970). The scientists continued observations on the previously constructed reefs, reporting significant deterioration of the car bodies on the Jacksonville reef, which had been in place for two years. The project team also introduced a mail survey targeting boat captains to estimate fishing pressure, catch-per-angler-hour values, and overall harvest values around the artificial reefs (Stone et al., 1970).

From 1969 onward, there was a concerted effort to communicate the benefits of tire reefs in mass media actively. Stone participated in interviews, and journalists were eager to publish his insights, as the topic appealed significantly to readers passionate about fishing and marine conservation. Stone unequivocally endorsed tires as the preferred material to build reefs. Tires were portrayed as advantageous because they facilitated marine growth, exhibited greater longevity compared to car bodies, and were considered easy to work with. The research team also actively promoted their engineering solution for tire reefs. They invited local reef-builder groups, encouraging them to reach out for information on the technical approaches to tire reef construction (Seward, 1969).

The construction of artificial reefs was portrayed in journalist writings as a beneficial endeavor. For instance, in a commentary dedicated to the research program, the outdoor editor of the Palm Beach Post Buckow wrote:

A big amberjack hovers over the bow of a sunken ship; a pair of groupers share the interior of an old automobile; a fat grey snapper peeks out from the darkness of a length of concrete culvert; tropical fish flit in and around a unit of auto tires resting on the bottom. This is what it’s like down under, on the artificial reefs that stretch along the Atlantic Seaboard from New York to Miami (Buckow, 1969b, p. D6).

It is possible to claim that such representation in mass media was an important factor that determined people’s involvement in reef construction. These waste reefs were depicted as lively places full of fish.

In 1970, Stone and colleague Chester Buchanan published an article titled ‘Old Tires Make New Reefs’ in the journal *Underwater Naturalist, The Bulletin of the American Littoral Society*, published by the Sandy Hook laboratory. The authors recommended tire module types and introduced a model of collective action that would later serve as a blueprint for the construction of numerous tire reefs:

Successful reefs are the result of continuing efforts by many individuals and groups in a community. This is perhaps the best reason why tires will prove to be particularly effective in building reefs. You can construct single and multiple tire units in any vacant lot and then distribute them to marinas or other waterfront areas where fishermen can easily pick them up and carry them out to the reef site on any kind of boat — private, charter or party. If many boats take a few tires out every day the reef will quickly grow to be a large and effective fish haven (Stone & Buchanan, 1970, p. 28).

The suitability for transport by small boats was a crucial factor influencing the use of tires, as initially, reefs were built by small groups of fishermen and non-profit organizations.

Stone’s credentials as a scientist were a very important factor in determining the persuasive force of his claims in mass media. For example, Ed Buckow, the Outdoors editor of the *Palm Beach Post* represented Stone as: ‘The biologist in charge of the U.S Bureau of Sports Fisheries five-year research project’ (Buckow, 1967a, 1967b). Journal *The Rotarian* goes even further and calls him the “US government authority of sunken junk” (*The *Rotarian, 1977). Holding the leading position in the government-funded research project made Stone notable and allowed his claims to be transformed into a collective action.

In 1971, certain aspects of artificial reef research previously managed by the Bureau of Sport Fisheries and Wildlife were transferred to the National Oceanic and Atmospheric Administration (NOAA). In 1970, President Nixon had established the NOAA within the Department of Commerce, bringing together several specialized agencies, including the Bureau of Commercial Fisheries, the Weather Bureau, and the Coast and Geodetic Survey. This merger aimed to create a more unified, ecosystem-centered approach to studying the oceans and atmosphere. The creation of NOAA also aligned with the goals of the National Environmental Policy Act (NEPA) of 1969, which required federal agencies to consider the environmental impacts of their actions. By transferring responsibilities like fishery management and environmental data services to NOAA, the move sought to enhance environmental protection and encourage more informed, public-inclusive decision-making at the federal level (Carter, 1970, 1970a). Consequently, in 1972, Richard Stone and Chester Buchanan were reassigned from Sandy Hook to the Beaufort Marine Laboratory in North Carolina, marking a shift in the location of artificial reef research (Weeks, 1972).

The transfer of the artificial reef research to NOAA coincided with the development of marine environmental science and a more ecosystem-centric approach. This transition is particularly evident in the changes to the working methods of the Stone team following their move to the new organization. Under USFWS, the primary method for artificial reef research focused on quantitative fish counts around the reef, which enabled the evaluation of effects on the targeted resource. In contrast, NOAA’s influence shifted the focus toward a stronger emphasis on assessing environmental impacts. However, the researchers’ deeply ingrained resource-centered perspective constrained their ability to fully exploit the potential benefits of this new approach. The new organization inherited not only the personnel but also their prior results and, equally significant, the cooperation agreements in which the Stone team had committed to finding solutions for the use of tires.

In Beaufort, the team embarked on an activity to produce some laboratory-controlled data about the environmental safety of tire reefs through fish toxicity experiments. The experiment involved two fish species known to inhabit artificial reefs: pinfish (*Lugodon rhomboides*) and black sea bass (*Centropristis striata*). Forty specimens of each species were placed in two 2000-L fiberglass tanks, each containing six used tires. Additionally, two similar containers with 40 specimens in each, but without tires, were used as control environments. After 21 days, 10 fish from each tank were sampled for zinc, organochlorine pesticides, and polychlorinated biphenyls (Stone et al., 1973, 1975). During sampling, the researchers had to remove the tires from both tanks to access the fish. Upon their return, a change in the behavior of the fish was noted in both containers. The black sea bass began to die and by day 38, all of them were dead. Conversely, no mortality was observed in the pinfish tank. Given that the analyses of tested toxicants did not indicate toxins within the fish tissues, the researchers put forth a hypothesis that the mortality was due to elevated organic content in the tank from accumulated food and fecal material inside the tires. To validate this hypothesis, the researchers duplicated the seabass experiment but this time with holes cut into the tires, thus enhancing water circulation. During this replicated experiment, the observed mortality was low. The tires were thus deemed to have no detrimental effects on pinfish and black seabass and to be suitable material for reef construction in many situations (Stone et al., 1975).

The design of this experiment illustrates that, despite changes in organizational structure, the scientists’ perspectives evolved more slowly, and it took time for their approaches to become fully ecosystem oriented. Although the goal had shifted to clarifying environmental impacts, the absence of ecosystem concepts in the team’s thinking is evident in this experiment. The scientists had not considered concepts such as uptake mechanisms and the upward movement through food chains. Pollutants typically first appear in organisms directly exposed to the pollutant, such as barnacles and other fouling organisms, rather than in fish, which have no direct contact with the tires and consume food from external sources. However, in this case, the scientists chose to measure pollutant levels in fish, as their primary concern was to assess the impact of such actions on a natural resource—namely, fish. Additionally, it is noteworthy that Stone seemed reluctant to accept the unfavorable results of the experiment, which were detrimental to the use of tires, and made efforts to overturn these findings through further experimentation.

In 1972, Stone participated in the Florida Aquanaut Research Expedition, organized by NOAA (Springer, 1972; The Miami News, 1972a), which established a tire reef located within 25 m of a natural patch reef in Biscayne National Monument, Florida. Fish counts and photography were used to monitor the development of both the artificial and natural patch reefs. By the conclusion of the research in August 1974, the artificial reef was treated entirely with Rotenone piscicide to kill the fish. The dead fish were collected to determine the standing crop and provide a baseline for evaluating the results of the earlier fish counts (Stone et al., 1979). The scientists concluded that, from an ecological perspective, the function of artificial reefs did not differ from that of natural reefs. This experiment underscores a striking blend of ecosystem-centered goals and resource-centered assumptions. Although the overarching objective was to assess differences in the ecological functioning of natural and artificial reefs, the sole indicator of ecosystem health chosen-fish numbers-was a fully resource-centered measure.

Stone actively published guidance documents on building reefs from tires. He was the lead author of the US Environmental Protection Agency report *Scrap Tires as Artificial Reefs* in 1974. The report focused on the economic aspects of constructing and deploying tire reefs, including extensive cost calculations for tire reef designs. The report also includes some data for anglers’ catches over the tire reefs. The document concludes that scrap tires, available at no cost, could be assembled into units and transported to reef sites at a moderate cost. Once on the seabed, they would serve as additional habitats for various fishes and invertebrates. According to the report, this approach also presents a beneficial use for tire waste: improving habitats instead of contributing to environmental issues like landfill pollution or mosquito breeding grounds (Stone et al., 1974). The report estimates that nearly one billion tires could be used for artificial reefs along the US East Coast (R. Stone et al., 1974). In the same year, Stone co-authored the NOAA report titled *How to Build Marine Artificial Reefs,* endorsing using tires (Parker et al., 1974). These two reports set out the environmental claim that waste tires made ideal artificial reef materials.

The artificial reef program in Beaufort officially ended in 1975 when Stone was reassigned to the NOAA Washington office (Wolfe, 2000). He was promoted to Deputy Acting Director of the Office of Marine Recreational Fisheries. His job included overseeing the coordination of artificial reef activities conducted by the agency and spearheading the formulation of artificial reef policy. Additionally, Stone served as the primary government liaison and consultant for states and foreign entities keen on engaging in artificial reef development (Smith et al., 2018). He played a central role in developing the National Artificial Reef Plan and supervised the creation of the NOAA artificial reef database (R. Stone, n.d.).

## Corporate bargain

Around 1970, Stone and his team started activities to persuade significant non-state actors to become involved in tire reef-building. These endeavors ultimately led to connections with the Goodyear Corporation. Richard Stone had been invited to present his tire reef concept to the company’s research and development division (Tolley, 1981). Goodyear’s Vice President, Thomas Minter, acknowledged the impact of Stone’s presentation in 1974 stating, ‘When Dick Stone dropped in to talk about reefs with some of our people about four years ago, he struck a responsive chord.’ (Minter, 1974). Subsequently, Goodyear emerged as a prominent player, contributing to the development and implementation of tire reefs.

Numerous interest groups dedicated to reef-building in Florida sought assistance from Goodyear, emerged in Florida. The real estate developer Deltona Corporation constructed the Marco Island artificial reef, with an estimated 10,000 tires, with vital assistance from Goodyear, which contributed machinery and the requisite quantity of tires (Grahame, 1972; Miller, 1962). Similarly, the Naples Cruise Club, led by two retired Goodyear engineers, undertook the construction of a fishing reef of tires off the Naples coast in Collier County. This venture benefited from substantial support from Goodyear (Richardson, 1973). In Lee County, a non-profit reef-building organization established by local fishermen’s clubs received direct consultations regarding reef design from Stone and enjoyed comprehensive backing from Goodyear (Taylor, 1973).

Broward Artificial Reef INC (BARINC) became the inaugural officially registered non-profit entity in the US dedicated to the construction of artificial reefs (Wilson, 1968). A consortium of individuals who shared a mutual interest in trophy fishing founded BARINC under the leadership of party boat captain Virgil Osborne. In 1968, BARINC successfully constructed an experimental reef of 400 concrete erosion control structures known as ero-jacks near Fort Lauderdale (Wilson, 1969). They had planned to construct an artificial reef extending over a three-mile expanse, which would be the most extensive project of its kind worldwide (Wilson, 1968, 1969). Still, it soon became apparent that ero-jack construction was prohibitively costly. After becoming acquainted with Stone’s initiatives and establishing connections with Goodyear, BARINC shifted its strategy to construction with tires (Fort Lauderdale News & Sun-Sentinel, 1971; McIntosh, 1974).

In the spring of 1971, BARINC president McIntosh presented the concept of a tire reef to the Broward County Pollution Control Board. During the presentation, he underscored that this approach would alleviate an air pollution issue caused by the fires in the county dump, where tires were accumulating. He also noted that Goodyear expressed their support for this method of tire disposal. Following the presentation, the commission reached a consensus and ‘strenuously recommended to the county and city governments that they actively engage in constructing an artificial reef’ (*Fort Lauderdale News and Sun-Sentinel*, 1971b). The active involvement of officials would have been unattainable without the prior recommendations of the artificial reef team advocating the construction of tire reefs.

By the end of 1971, the financial backers of BARINC were in place. Goodyear donated a tire compaction apparatus and tires and extended financial support, and the Port Everglades authorities made a philanthropic donation of two acres of land for the storage and compaction activities associated with the tires. Broward County formally engaged in the BARINC project to oversee the meticulous preparation of tire modules, their concrete filling, and subsequent loading onto a barge (Wilson, 1971, 1972a). The first tire deposition on the Fort Lauderdale Osborne reef occurred on April 22, 1972 (Wilson, 1972a, 1972b, 1972c, 1972d). This event involved over 150 vessels and was orchestrated as a grand celebratory event, presided over by the Goodyear blimp (Wilson, 1972c).

At the begining, BARINC actively sought contributions to fund the hiring of boats and barges to transport the tire modules to the reef site (Wilson, 1972d). However, in 1973, the organization found itself unable to cope with the escalating volume of tires requiring disposal, and the County Commission for Further Development decided to assume control of the project. As part of their plan, the county intended to ban tire disposal at their landfill after the project was fully operational (Fort Lauderdale News, 1973).

In 1974, reef construction expanded to an industrial scale, with an astounding 200,000 tires deposited on the reef during a single deployment event. Such large-scale operations became a regular occurrence. Concurrently, Broward County extended financial support, procured new compactors and hole-punching machinery, and employed nine full-time personnel to create tire bundles (Wilson, 1974). The county had succeeded in establishing the largest artificial reef in the world (Wulf, 1974).

Yet county officials soon began openly criticizing the project as it became increasingly apparent that the reef failed to attract marine life. In February 1975, County Commissioner Anne Kolb attempted to halt the project but was unsuccessful in her efforts. The supervisor of the tire processing site, O. Riggle, offered the following remarks on the matter: ‘Whether the reef is working or not, the system is doing the county service. The tires would have to be disposed of somehow, and this way there’s no pollution’ (Faherty, 1975). By 1979, when the project was ultimately terminated, a whooping 4 million tires had been dropped into the ocean (Thompson, 1979).

Over the following two decades, numerous tire reefs were constructed along the coast of Florida, South Carolina, and North Carolina. In the 1980s and 1990s, there was an active phase of tire reef construction in New Jersey and Delaware, with certain projects exceeding the size of the Osborne Reef (Angst & Angst, 1992; NOAA Office for Coastal Management, 2022). The NOAA artificial reef database contains data about nearly 750 tire reefs in the Atlantic states alone (NOAA Office for Coastal Management, 2022).

## The creative engineering of tire reefs

Returning to the Marathon Reef, which began this article, we see that the conjunction of fishing interests and waste management was in full force in 1977. The depiction reveals a sizable festive gathering, with most Marathon residents actively participating. Inspiring classical music is playing, and the screen shows a large fleet of vessels led by the Goodyear blimp going to sea. The camera shows the blimp up close; the captain of the coastal guard vessel says on the radio that the target area is clear, and the blimp drops down the first tire. The voiceover says, “This is the place, folks”, and the people on the boats start throwing their tire cargo overboard (McGowan, 1977). Everything seemed to be a success.

Yet by 1979, it became evident that the Marathon Reef had failed. Ocean currents and waves disrupted the reef, destroying surrounding coral formations and flattening turtle grass habitats crucial for the foraging of grouper, snapper, and baitfish. Only 100 out of the 800 initially deployed bundles were located at the designated site two years after the construction. The migration of modules introduced a palpable harm to the very resources the builders sought to protect (Associated Press, 1979).

The effects of Osborne Reef, built by BARINC, were even worse due to its much larger size. The tire bundles underwent gradual disintegration, tires became mobile, and the reef transformed into a huge subaquatic dump. It undulated in the oscillations of the waves, thereby pulverizing the adjacent reefs and the encompassing marine life (Sherman & Spieler, 2006). At the beginning of this century, some expensive and technically complex measures were taken to remove a part of the tires from the reef (Flesher, 2015); however, most remain on the ocean bottom.

The case of tire artificial reefs fits well with Hannigan’s ideas about how environmental problems are discursively created. Stone, as leader of the USFWS / NOAA artificial reef research program, framed the problem of artificial reef construction as tied to the problem of tire waste disposal. He had scientific authority and was responsible for validating claims. The reports produced by Stone played up the value of waste tires as a waste material and played down other potential reef materials, even ones like concrete, that appeared to show better scientific results. At the same time, he served as a populariser who advocated for making reefs out of tires to a broader audience. Media attention and appropriate framing of his claims in the press were also the results of his authority: mass media dramatized the proposed solution in symbolic and visual terms. Stone was also responsible for the economic calculations related to the approach proposed and was able to prove that building reefs from tires is economically beneficial. Stone successfully enlisted substantial institutional players, and waste tire reefs became a reality.

An environmental claims-making approach gives insights into how a scientist or group of scientists, like the ones who worked on the artificial reef program, create and disseminate knowledge, which is then acted on by others. Their claims-making activities lay the groundwork for a shared understanding of the problem and its potential solutions. In the case of artificial reefs, this meant that tires were conceptualized and communicated as appropriate, and even preferred, construction material to address two problems that, on the surface, had nothing to do with each other. Claims-making brought together the desire for fishing reefs and the need to dispose of waste tires as creative environmental engineering.

## Data Availability

Not applicable.
